# Supplementary material for: Remarkable performance recovery in highly defective perovskite solar cells by photo-oxidation
Source: J Mater Chem C Mater. 2023 Feb 17;11(24):8007–17. doi: 10.1039/d2tc05077c (PMC10286220; doi:10.1039/d2tc05077c)
Supplement: TC-011-D2TC05077C-s001 [file TC-011-D2TC05077C-s001.pdf]

Electronic Supplementary Material (ESI) for Journal of Materials Chemistry C.
This journal is © The Royal Society of Chemistry 2023

Remarkable performance recovery in highly defective perovskite solar cells by photo-oxidation

Katelyn P. Goetz[1], Fabian T. F. Thome[3], Qingzhi An[1], Yvonne J. Hofstetter[1,2], Tim Schramm[1,2], Aymen Yangui[4], Alexander Kiligaridis[4], Markus Loeffler[5], Alexander D. Taylor[1], Ivan G. Scheblykin[4], Yana Vayznof[1,2*]

1. Chair for Emerging Electronic Technologies, Technical University of Dresden, Nöthnitzer Str. 61, 01187 Dresden, Germany
2. Leibniz-Institute for Solid State and Materials Research Dresden, Helmholtzstraße 20, 01069 Dresden, Germany
3. Kirchhoff Institute for Physics, University of Heidelberg, Heidelberg, Germany
4. Chemical Physics and NanoLund, Lund University, Lund, Sweden
5. Dresden Center for Nanoanalysis, Technical University of Dresden, Dresden, Germany

Corresponding Author

*Prof. Dr. Yana Vaynzof, yana.vaynzof@tu-dresden.de

Table of Contents

1. Solar Cell Characterization 3
2. Light and Atmosphere Dependence of Solar Cell Performance 6
3. Composition-Dependence of Treatment Response 9
4. Cross-sectional SEM Imaging 12
5. Time-dependent Degradation of 2.96 Films 12
6. X-ray Photoelectron Spectroscopy Methods and Results 14
7. Photoluminescence Spectroscopy Methods and Results 15

1. Solar Cell Characterization

The typical effect of multiple JV scans is shown in Fig. SI1 and SI2, where the open-circuit voltage (V_{OC}) increases under repeated measurements and the short-circuit current (J_{SC}) decreases slightly. Fig. SI3 shows the external quantum efficiency characteristics for each stoichiometry, reference and treated. Dark JV curves were measured from 1.2 V - -0.3 V and back with a step size of 0.05 V and a dwell time of 0.1 s at each step (Fig. SI4).

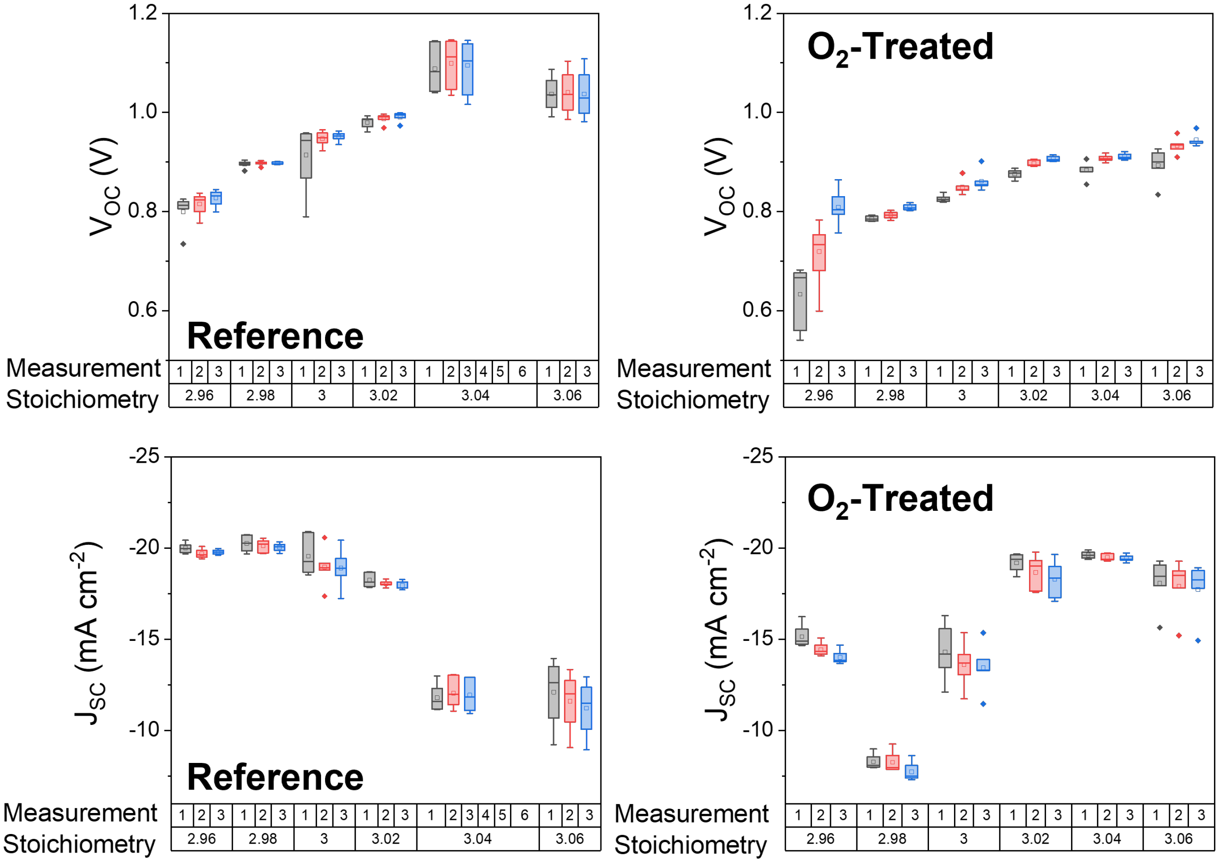

Figure S1: The effect of multiple scans on the open-circuit voltage (V_{OC}) and short-circuit current density (J_{SC}) of a batch of solar cells. All stoichiometries are shown for reference cells and cells treated with oxygen and light, for three measurements.

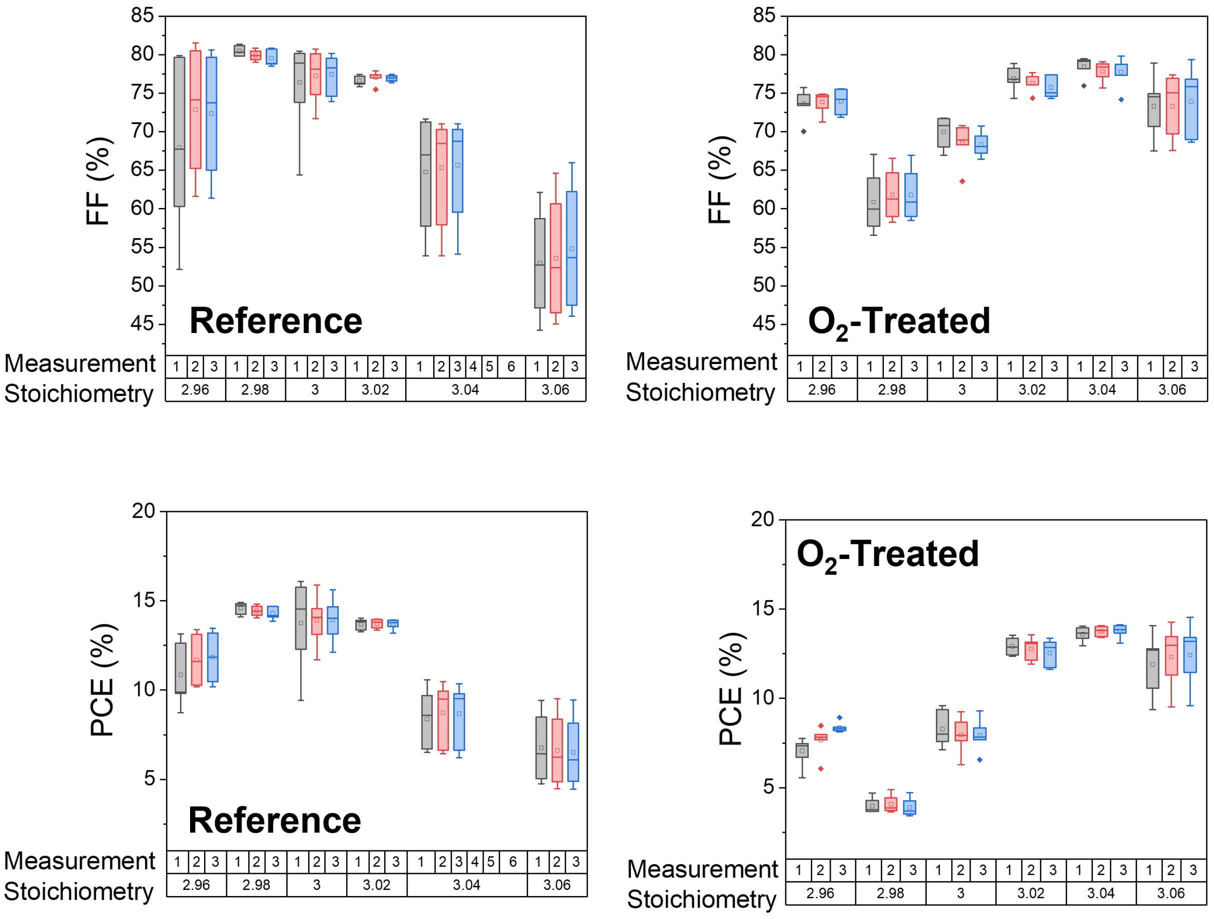

Figure S2: The effect of multiple scans on the fill factor (FF) and the power conversion efficiency (PCE) of a batch of solar cells. All stoichiometries are shown for reference cells and cells treated with oxygen and light, for three measurements.

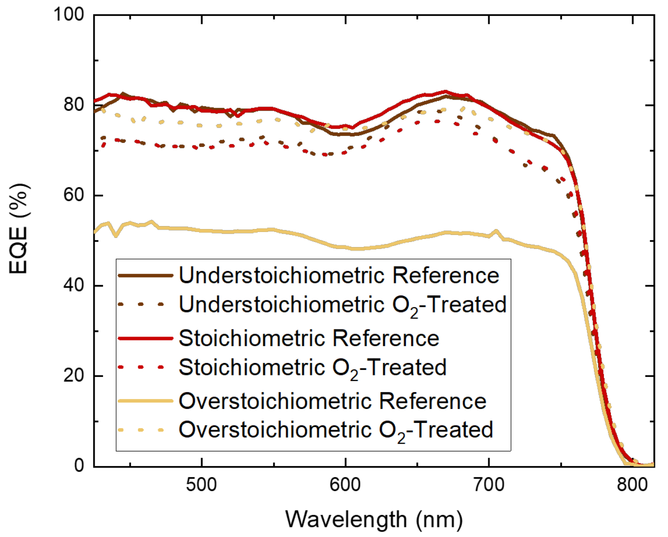

Figure S3: Example EQE of understoichiometric (MAI:Pb(OAc)$_2$ = 2.96), stoichiometric (MAI:Pb(OAc)$_2$ = 3.00), and overstoichiometric ((MAI:Pb(OAc)$_2$ = 3.06) solar cells. The understoichiometric and stoichiometric show degradation, while the overstoichiometric show recovery.

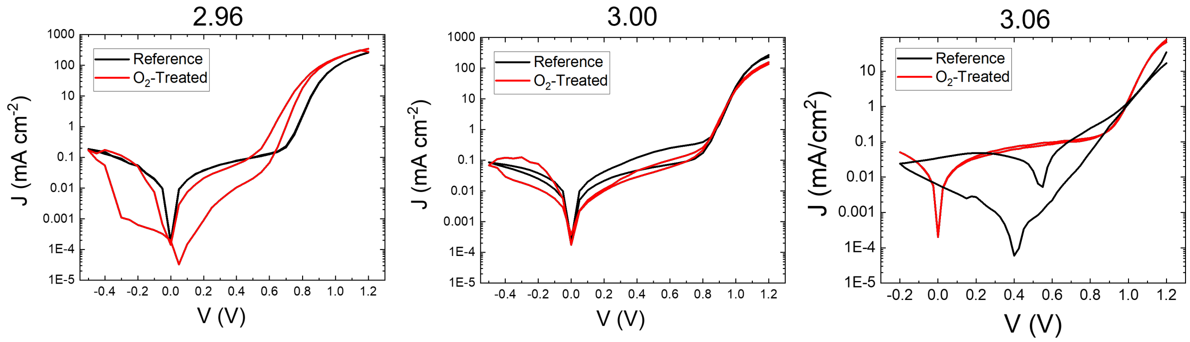

Figure S4: Example dark JV curves for understoichiometric (MAI:Pb(OAc)$_2$ = 2.96), stoichiometric (MAI:Pb(OAc)$_2$ = 3.00), and overstoichiometric ((MAI:Pb(OAc)$_2$ = 3.06) solar cells.

2. Light and Atmosphere Dependence of Solar Cell Performance

To test whether the observed degradation was due to oxygen, light, or the combination of both, we stored solar cells under a light and nitrogen environment, and under an oxygen and dark environment. Example J-V curves for the 3.06 stoichiometry are shown for each experiment in Fig. S5, and the device statistics are shown in Fig. S6 (treatment = dark, oxygen) and Fig. S7 (treatment = nitrogen, light). Experiments were performed in the same sample holder as the light and oxygen treatment, with only the gas type or lighting condition changed. Samples treated in the dark under an oxygen atmosphere or treated under AM 1.5 simulated light and a nitrogen atmosphere do not show a significant difference from the reference cells left in the dark in a nitrogen glove box. Extra sample-to-sample variation is expected, in particular, at the 3.04 stoichiometry (note that Fig.1 shows a wider range of values at this stoichiometry for the reference cells than others). We believe this accounts for the difference between the reference cells (nitrogen, dark) and treated cells (nitrogen, light) shown in Fig. S7. Especially significant is the consistent presence of hysteresis and low current density for overstoichiometric samples. Healing only takes place for samples exposed to oxygen and light.

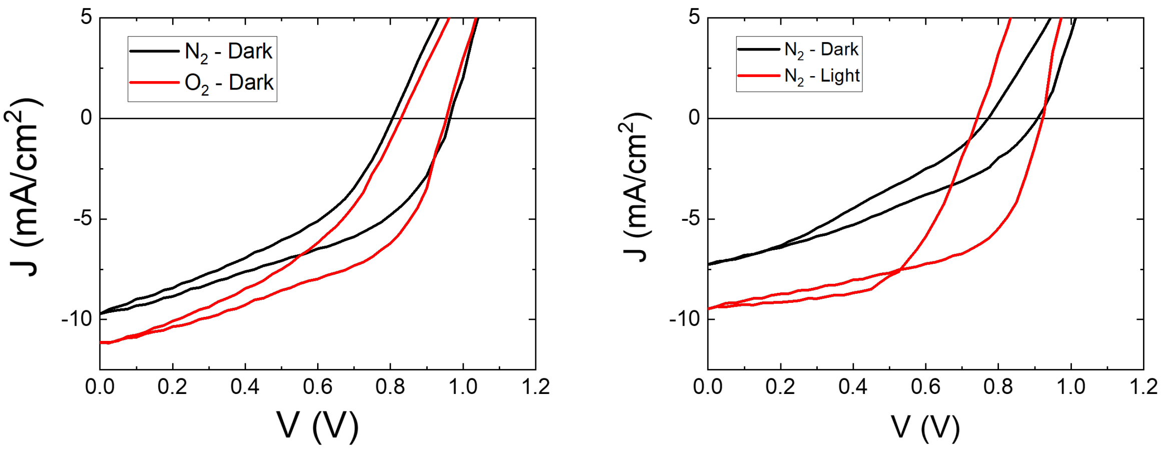

Figure S5: Example JV curves for reference films and films treated with oxygen in the dark. All curves show low current density and significant hysteresis.

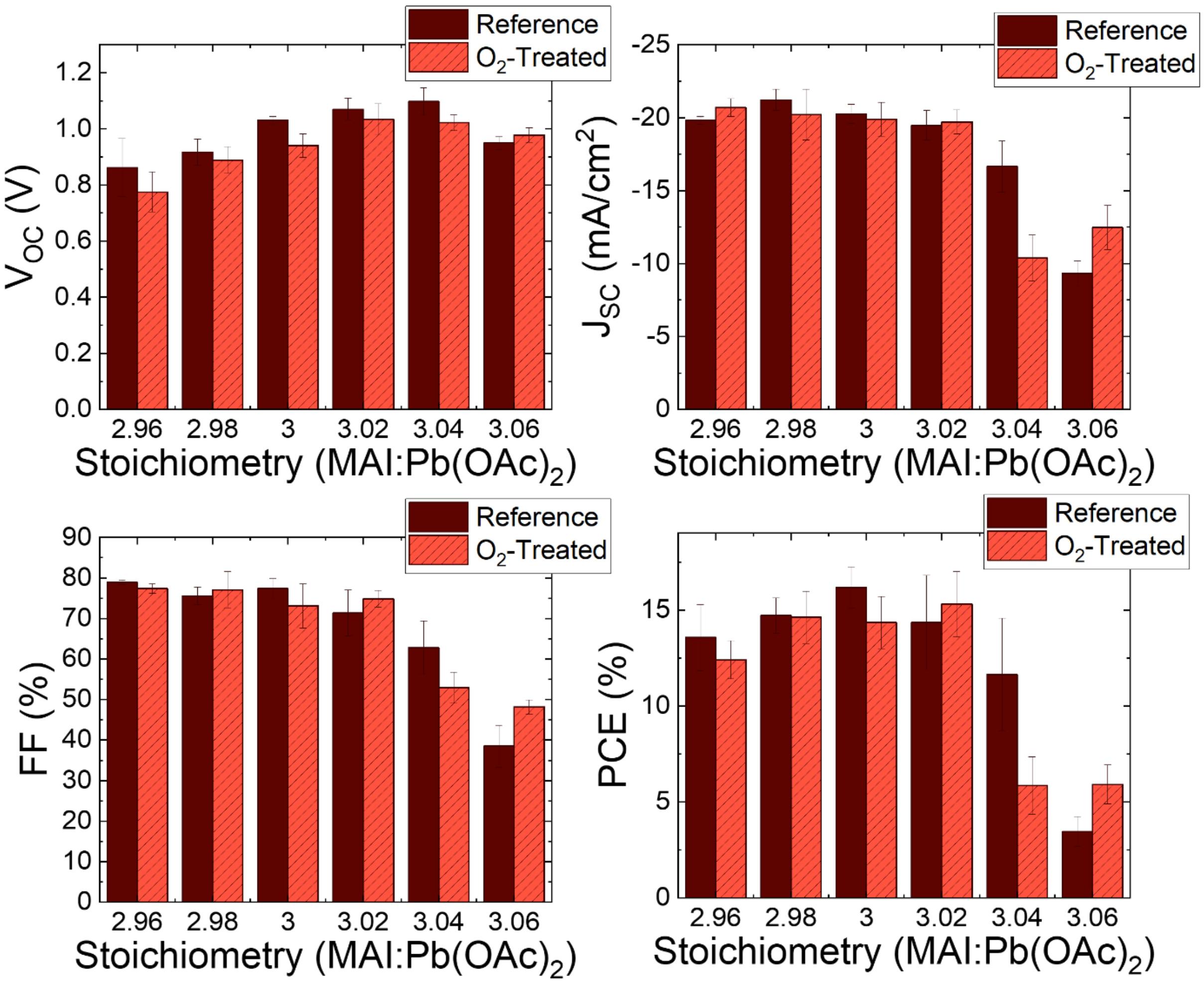

Figure S6: A comparison between a reference batch of samples (dark, nitrogen storage of the perovskite films) with samples treated with oxygen in the dark.

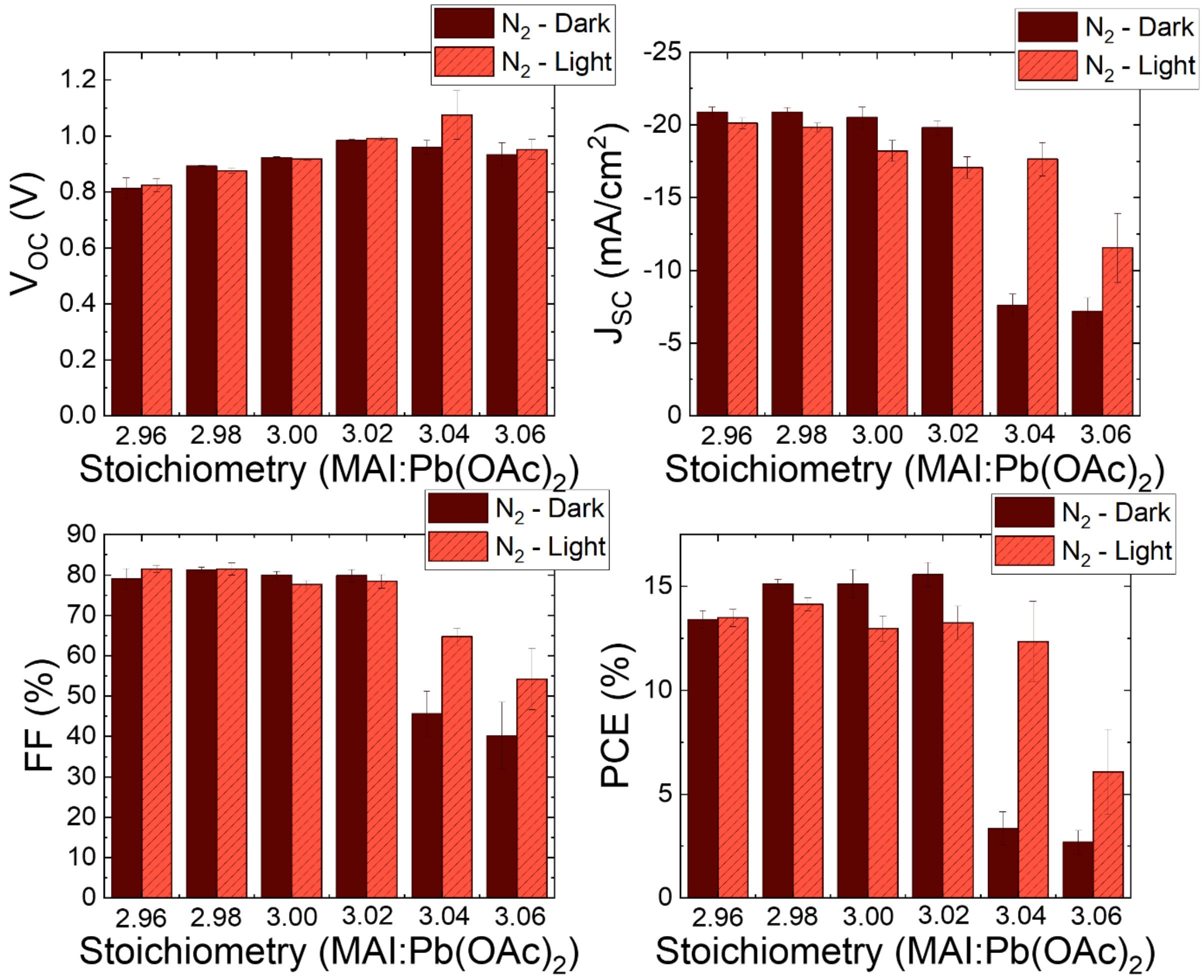

Figure S7: A comparison between a reference batch of samples (dark, nitrogen storage of the perovskite films) with samples treated with nitrogen and light.

3. Composition-Dependence of Treatment Response

PV characteristics for the triple-cation perovskite solar cells are shown in Fig. SI8, with an example JV curve shown in Fig. S9. Likewise, the MA-free cell characteristics are depicted in Fig. S10 and Fig. S11. For the MA-free cells, the x = 0.93 reference cells are expected to be an outlier.

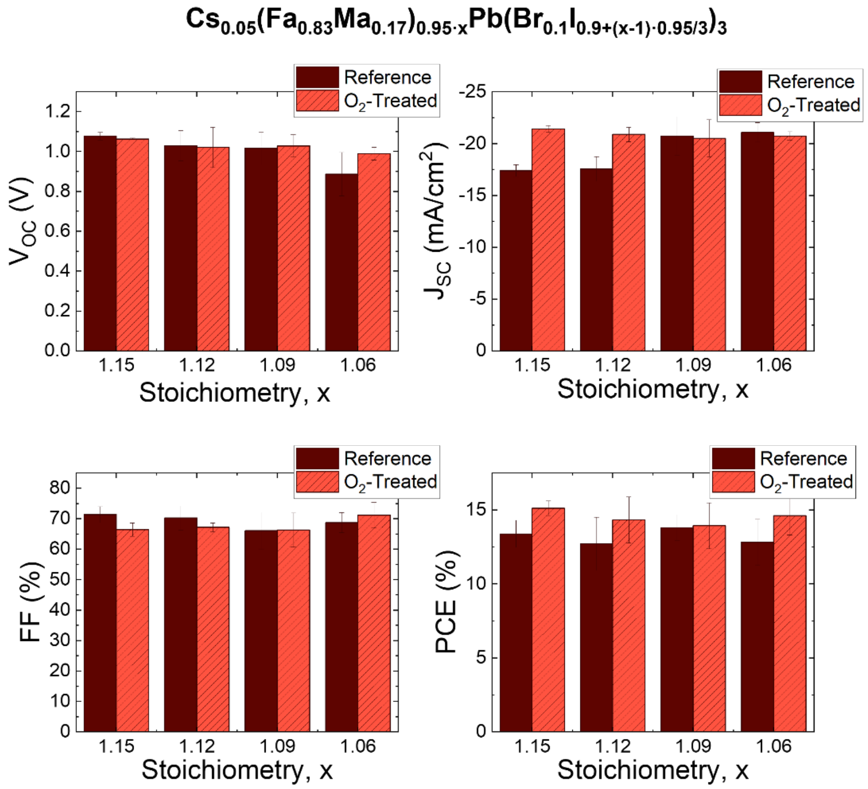

Figure S8: The effect of light and oxygen treatment on triple cation solar cells. 1.15 is the most overstoichiometric.

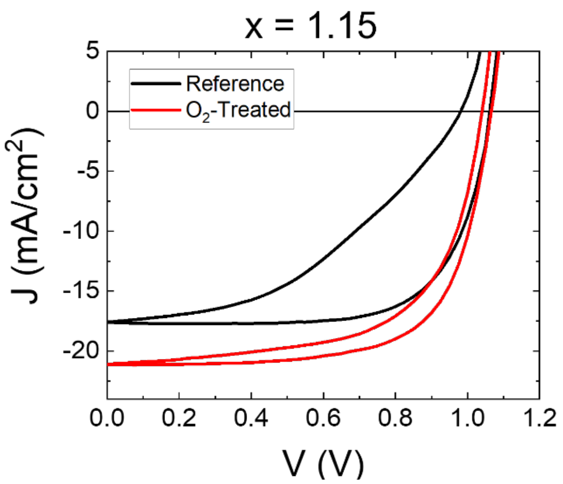

Figure S9: JV characteristics for x = 1.15 stoichiometry. The hysteresis and short-circuit current density both improve upon treatment with oxygen and light.

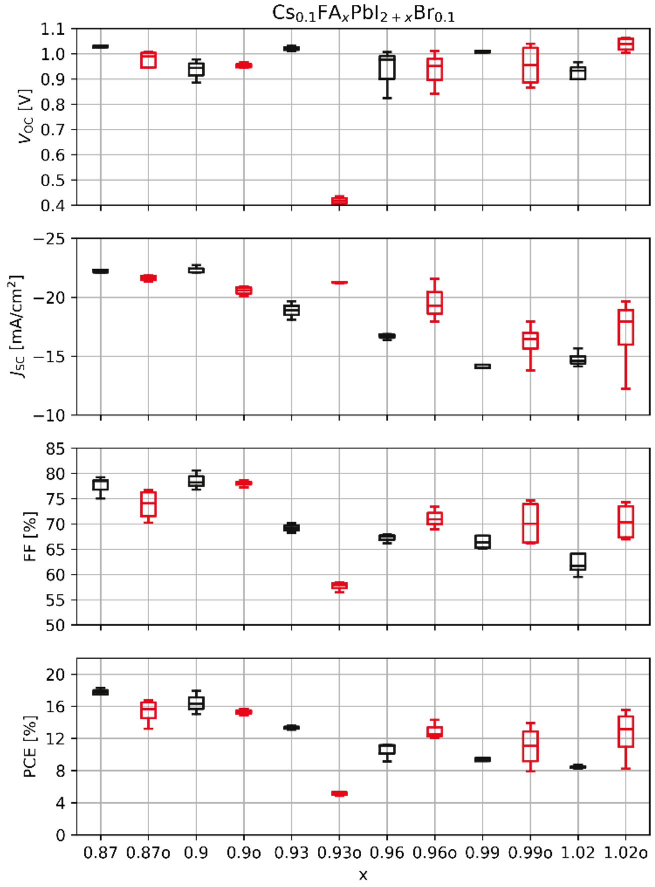

Figure S10: Photovoltaic characteristics for MA-free solar cells, with and without treatment with oxygen and light. Black are the reference cells, red or the oxygen-treated cells. The 0.93 stoichiometry is likely an outlier.

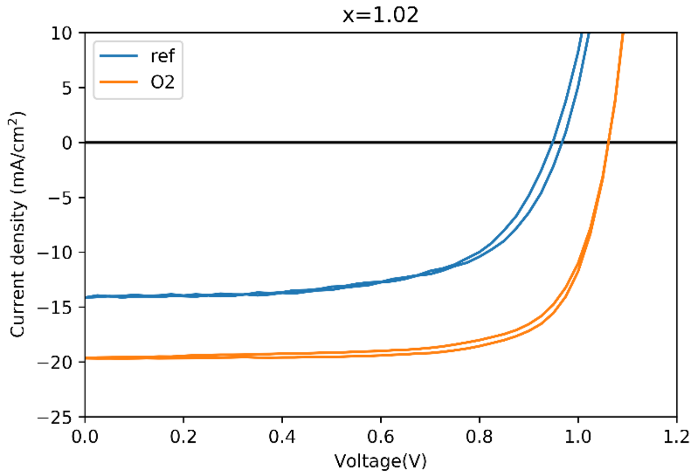

Figure S11: JV characteristics for treated and reference overstoichiometric films. The short-circuit current density improves for the oxygen-treated cells.

4. Cross-sectional SEM Imaging

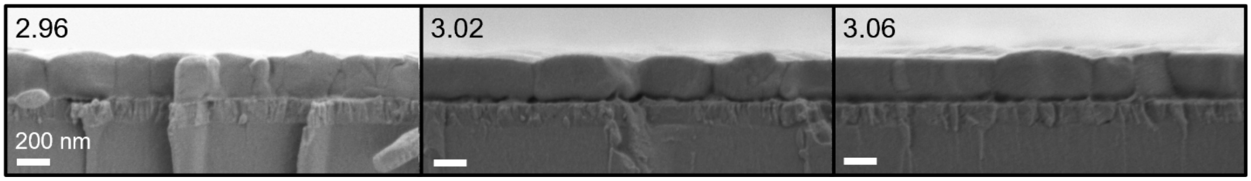

Figure S12: Cross-section SEM images of films made from 2.96, 3.02, and 3.06 stoichiometry precursor solution following exposure to oxygen and light.

5. Time-dependent Degradation of 2.96 Films

The time-dependent degradation of the understoichiometric films is shown in Fig. S12. The J_{SC} (Fig. S12b) decays continuously from approximately -20 mA/cm^2 at 0 hrs to -8 mA/cm^2 at 10 hours treatment with light and oxygen. The SEM shows surface roughening as early as 2.5 hours into the degradation process, with no cross-sectional decay at 15 hours (Fig. S12c). The XRD shows the presence of only the 002 and 004 peaks at 0 hours, with the gradual emergence of the 110 and 220 peaks over time, as the 002/004 decrease in intensity (Fig. S12d).

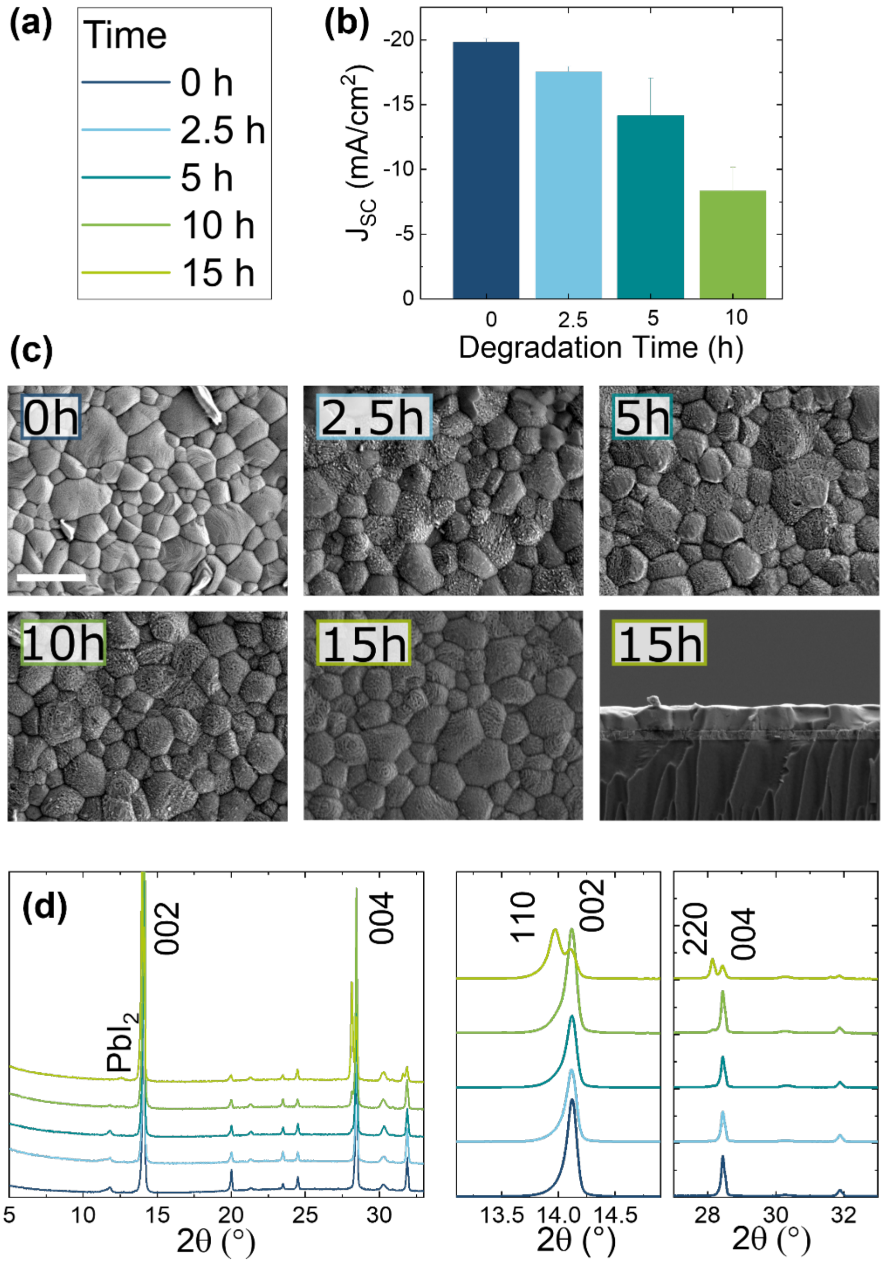

Figure S13: The time-dependent degradation of understoichiometric cells. a) The legend. b) The degradation of the short-circuit current, showing gradual decay. c) SEM of degraded understoichiometric films. The scale bar is 500 nm. All images are the same magnification. d) X-ray diffraction of understoichiometric perovskite films.

6. X-ray Photoelectron Spectroscopy Methods and Results

XPS measurements are shown in Fig. S13.

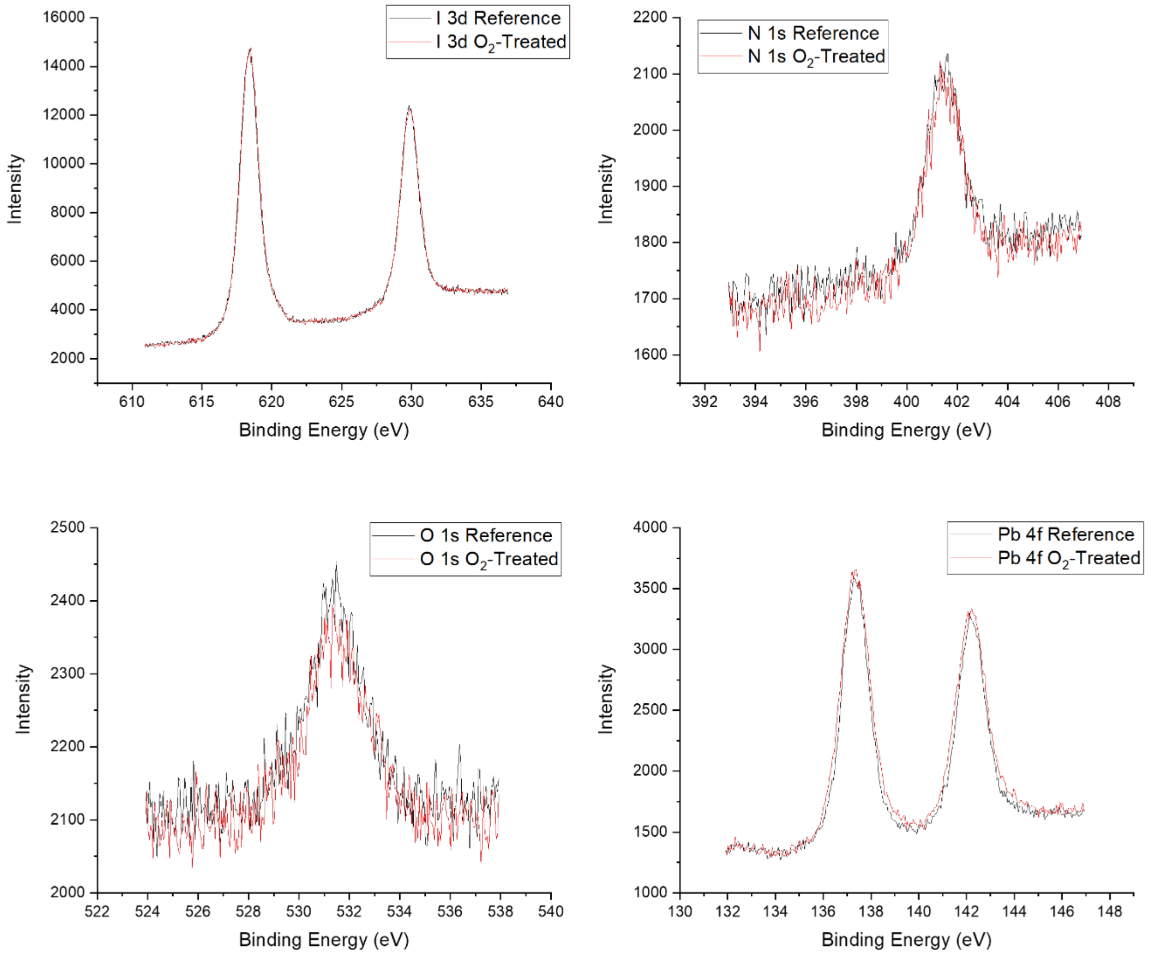

Figure S14: XPS spectra for oxygen, lead, nitrogen, and iodine.

7. Photoluminescence Spectroscopy Methods and Results

Steady-state PLQE measurements are shown in Fig. S14.

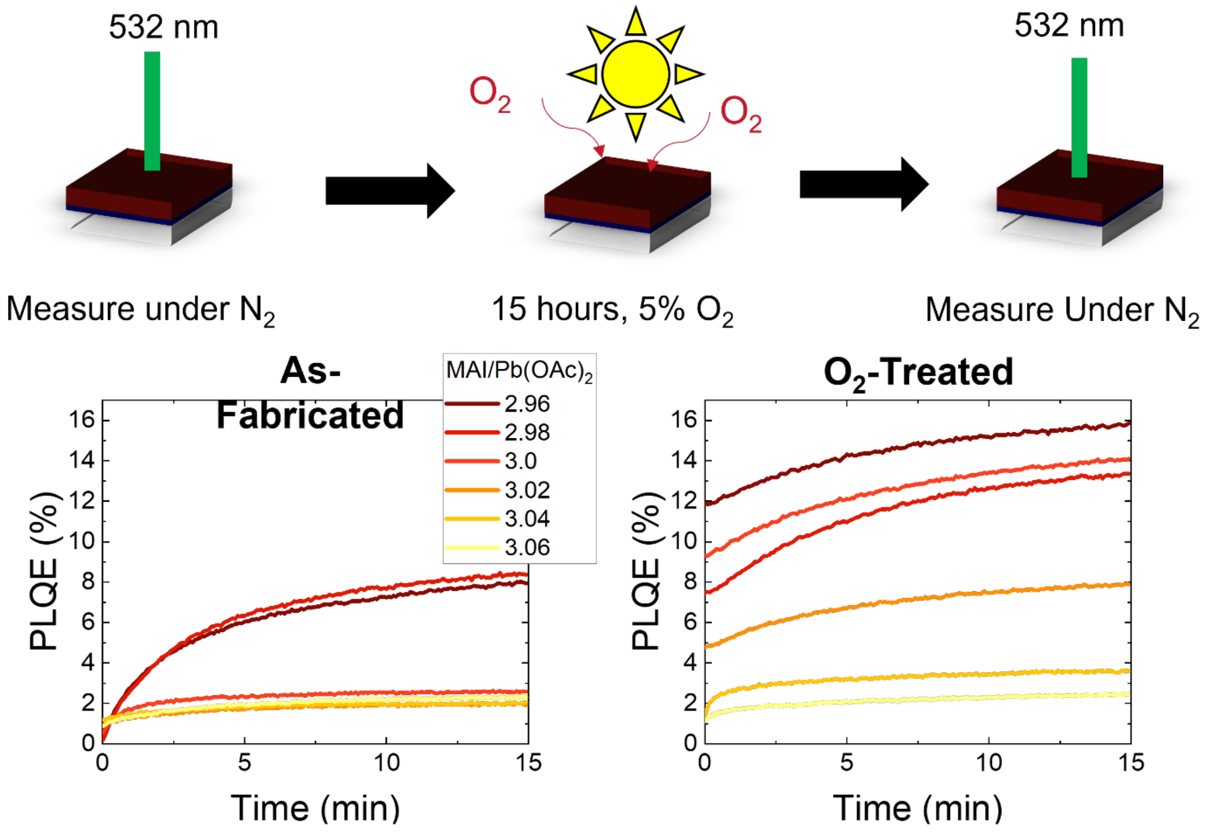

Figure S15: The steady-state PLQE of the as-cast and oxygen/light-treated perovskite films, for all stoichiometries.
